# ISOMED - A Stable ISOtope database of MEDiterranean marine food web components

**DOI:** 10.1038/s41597-025-05981-y

**Published:** 2025-11-11

**Authors:** Daniela Berto, Emanuela Fanelli, Salvatrice Vizzini, Federico Rampazzo, Zaira Da Ros, Seta Noventa, Tomaso Fortibuoni, Camilla Antonini, Giovanna Cilluffo, Geraldina Signa, Alice Premici, Roberta Bardelli, Saša Raicevich

**Affiliations:** 1 Italian Institute for Environmental Protection and Research, ISPRA (Istituto Superiore per la Protezione e la Ricerca Ambientale), Brondolo, Mercato Orticolo 6, 30015 Chioggia Venezia, Italy; 2 Dept. of Life and Environmental Sciences, Università Politecnica delle Marche, Via Brecce Bianche, 60131 Ancona, Italy; 3 Department of Earth and Marine Sciences, University of Palermo, via Archirafi 18, 90123 Palermo, Italy; 4 National Inter-university Consortium for Marine Sciences, Piazzale Flaminio 9, 00196 Roma, Italy; 5 Italian Institute for Environmental Protection and Research, Via Ca’ Fornacetta 9, 40064 Ozzano dell’Emilia Bologna, Italy

**Keywords:** Ecology, Environmental sciences, Zoology

## Abstract

Marine food webs shape the ecological dynamics of energy flows and predator-prey relationships. Their study is crucial to understanding human impacts that are monitored within environmental policies, such as the European Marine Strategy Framework Directive. One of the most used methods to assess the structure and functioning of marine food webs relies on the use of carbon and nitrogen stable isotopes (*δ*^13^C and *δ*^15^N), which are particularly useful for a vast range of taxa whose diet composition may be impractical to evaluate through traditional approaches (i.e., stomach content analysis). However, such data are sparse and have not been consistently collected, making it often difficult to assess the status of marine food webs. Here, we present ISOMED, a georeferenced database of published *δ*^13^C and *δ*^15^N values and carbon and nitrogen elemental composition of basal sources of organic matter and consumers collected in the Mediterranean Sea. The information reported includes estimates for 4959 records. ISOMED provides a unique tool for investigating trophic interactions and energy flow within Mediterranean food web components.

## Background & Summary

The most recent policy developments in marine environmental protection show the emerging need to address the effects of anthropogenic pressures not only at species and habitat levels but also on the marine ecosystem as a whole^1^.

This is the essence of the ecosystem approach, which was formerly developed for sectoral policies (*e.g*., under fisheries management)^2^ and gained traction as a new paradigm to address the complex expressions of cumulative human-induced pressures by considering, among others, the structure and functioning of food webs. Marine ecosystems may provide essential ecosystem services for human well-being, including carbon sequestration, the presence of iconic wildlife^3^, and support for fisheries and Blue Growth^4^. Policies based on the ecosystem approach, like the Marine Strategy Framework Directive (MSFD)^5^, whose implementation spans beyond the EU marine waters (including the whole Mediterranean Sea, the Black Sea, the Baltic Sea, and the North-east Atlantic Ocean), require the development and operationalisation of food-web indicators and models^6^.

Different approaches can be used to investigate food web properties for this purpose^7^. For instance, data-based approaches can be applied to reveal natural processes using stomach content analysis and trophic markers such as stable isotopes and/or fatty acids. At the same time modelling techniques can enhance our understanding of food webs, providing a more comprehensive assessment of their structure, functioning, and dynamics. A further approach, in some cases bridging the two already mentioned, entails the use of indicators, either related to the whole ecosystem or to its components (see^8^).

Within the MSFD (according to Commission Decision 848/2017^9^), the assessment of marine food webs, carried out in the context of Descriptor 4, either for the primary criteria related to diversity and abundance of trophic guilds (namely D4C1 and D4C2) and the secondary criteria referring to the size distribution within guilds and productivity of guilds (namely D4C3 and D4C4) requires the assignment of species to guilds^6^. Other integrative approaches, i.e., those based on the marine trophic index (MTI^10^), consider changes in the mean trophic level of the catches and are proposed as “proxy indicator” for Target 14.2 of Sustainable Development Goal 14 (‘Life below Water’) (https://www.un.org/sustainabledevelopment/goal-14-life-below-water).

Key to supporting the implementation of these approaches is having a good understanding of the species’ roles within the food web and defining the species’ trophic position or level^11,12^. In this context, carbon and nitrogen stable isotopes (*δ*^13^C and *δ*^15^N) can be instrumental in allowing an integrative and robust assessment of species’ feeding ecology for a vast range of taxa whose diet composition may be impractical to assess or for whom data are scattered or inherently biased. Stable isotope analysis (hereafter SIA) is based on the predictable change (i.e., fractionation factor) in the isotopic ratios between a consumer and its prey. This factor is relatively low for carbon isotope ratios (theoretically +1‰ at each trophic level^13,14^), being larger for nitrogen (theoretically from +2.5 to +3.4‰ at each trophic level^15,16^). Nitrogen is thus commonly used as a proxy for the trophic level of the consumer, while carbon serves as a tracer of the sources of organic matter at the base of the food web that leads to the consumer. The fractionation factors can vary with feeding behaviour, trophic position, metabolism, body size or temperature^17–20^, suggesting that the assessment of this factor is crucial for food web reconstruction and experimental determinations are needed.

A comprehensive understanding of the isotopic composition of nitrogen and carbon sources utilized by primary producers is essential for the accurate application of stable isotope analysis (SIA) in food web research, as these baseline signatures are mirrored across the food web^15^.

Numerous reviews have been published on the application of stable isotopes to marine biogeochemistry^21^, plant and animal ecology^22–24^, food web reconstructions^25,26^, estimation of cumulative anthropogenic pressures through food web-scale indicators^27^, and further support the broader value of stable isotopes in ecological studies.

Here, we present a database (ISOMED) of *δ*^13^C and *δ*^15^N values, along with C and N elemental values, for basal organic matter sources and consumers in Mediterranean marine ecosystems, spanning the temporal range from 1997 to 2020.

The Mediterranean Sea hosts more than 17,000 marine species, with the highest rate of endemism globally (20–30%)^28^. Despite being a biodiversity hotspot, this basin is one of the most threatened in the world, being affected by multiple stressors, including climate change (warming, salinisation, and oxygen depletion), overfishing, and, in coastal areas, land-derived pollution, which are the major drivers^29^. The Mediterranean countries and institutions are lagging in the assessment of the ecosystem and food-web indicators for marine environmental protection, and it is necessary to progress quickly in support of the implementation of the Ecosystem Approach^30^.

In this context, the ISOMED database represents a valuable tool for estimating the spatial and temporal variability of isotopic data at the Mediterranean level, inferring the causes of such variability, and investigating the trophic position of a vast range of taxa. In addition, ISOMED is relevant for all researchers working on feeding ecology, marine food web structure and functioning, and environmental assessment. Being data georeferenced, ISOMED enables considering of spatial factors when identifying changes in food webs and exploring the links to possible effects caused by human-related (direct or indirect) disturbances (e.g., fishing activities, climate change, non-indigenous species, etc.). ISOMED is enriched with biological data on the size and weight of sampled specimens to allow users to factor out possible intra-specific size-based biological effects in modulating stable isotope values (i.e., due to ontogenetic shifts).

ISOMED enables access to spatially explicit stable isotope data on a vast range of basal sources of organic matter and consumer taxa of the Mediterranean Sea. The use of this information, in combination with other ecological data and modeling tools, will facilitate a more comprehensive understanding of marine ecosystem functioning and support the implementation of the ecosystem approach to protect sensitive ecosystems.

In addition, ISOMED can serve as a reference database supporting a consistent implementation of the MSFD and the Ecosystem Approach in the Mediterranean Sea, specifically for representing a reference database supporting a consistent implementation of the MSFD and the Ecosystem Approach in the Mediterranean Sea, particularly in relation to food web descriptor 4 and ecological objective 4, respectively. In particular, this database enables the consistent (Mediterranean-based) operationalization of the use of trophic guilds assessment (i.e., facilitates assigning species to trophic guilds), indicators based on trophic level (i.e., enables accurate trophic position estimates), and informs the construction of food web models concerning prey-predators linkages (e.g., supporting diet matrix estimates in mass-balance models, such as Ecopath with Ecosim).

## Methods

### Data sources

ISOMED gathers published and unpublished data of *δ*^13^C and *δ*^15^N values and elemental content (C and N) of basal sources of organic matter and consumers collected in the Mediterranean Sea. Published data refer to scientific peer-reviewed papers selected from the literature through a systematic search, whereas unpublished data correspond to the raw *δ*^13^C and *δ*^15^N data by the authors of the present paper. Data from open-access repositories were not included, as they are already easily accessible.

The literature search was carried out via Web of Science. We used multiple combinations of keywords and terms related to three specific data categories: stable isotope composition, trophic position, and the study area, according to the following search strings:

Stable AND isotope OR nitrogen isotope OR carbon isotope, Trophic level OR food, Mediterranean Sea.

After collecting 331 papers, we performed a pre-selection of the potentially relevant ones. We screened the full text and retained only those that: 1) reported original data for *δ*^13^C or *δ*^15^N in basal sources of organic matter and/or consumers, 2) referred to Mediterranean food webs, including those in nearby brackish waters (e.g., lagoons, estuaries), and 3) described the methodology used for sampling and isotopic analysis in sufficient detail to permit a quality assessment by authors who are experts in this field.

### Data extraction, cleaning, and formatting

The selected papers were carefully analysed to extract all relevant information describing *δ*^13^C and/or *δ*^15^N data in the Mediterranean Sea. We focused on the entire food web, from basal sources of organic matter (including sedimentary and suspended organic matter, as well as phytoplankton and macrophytes) to low-order consumers (e.g., meiofauna, mesozooplankton), high-order consumers, and top predators.

The extracted information was assembled into a single spreadsheet (Microsoft Excel), enabling the consolidation and standardisation processes.

All the information referring to a single sample was annotated in a single string. Specifically, each entry provides georeferenced *δ*^13^C and/or *δ*^15^N data for a single specimen, a group of specimens belonging to the same taxon, or a single sample of basal organic matter collected in the Mediterranean Sea. When available, the elemental content of C and N was also reported. The following criteria were adopted in order to include the information found in the dataset: (1) original data, (2) sample consistency (i.e., taxon consistency for aggregated samples of organisms, macrogroup consistency for basal sources of organic matter and low-order consumers), and (3) indication of the sampling location that at least enable its georeferentiation at basin/sub-basin level or with higher precision. Additionally, samples were included when sourced from fishermen or local markets as long as the authors deemed them reliable suppliers for obtaining suitable samples for their scientific research. In such cases, the coordinates of a randomly selected point in the fishing area were recorded.

When available, additional details on the samples, sampling sites, and timing were extracted and rationalised into pre-established metadata categories. The dataset structure consists of 35 columns (Table 1): 26 columns are dedicated to metadata variables concerning sample collection (e.g., sampling sites and timing), while nine columns are dedicated to isotopic characterisation. When no or ambiguous information was provided to fill the metadata fields, we entered “Not applicable” (i.e., na) in the cells. Each record was provided with a sequential number (column: ***Record number***) and full bibliographic reference (***Bibliographic reference***).

**Table 1:** ISOMED database structure.

Data Category	Parameter Short Name	Parameter Description
**DB compilation**	***Record number***	Record sequential numbers.
**Sample**	***Original sample name***	The term used by the authors in the source publication to denote the sample.
**Sample**	***Functional category***	Sample classification of basal sources of organic matter and low-order consumers into key functional macrocategories (i.e., Benthic diatoms, Epiphytes, Halophyte, Macroalgae, Meiofauna, Microplankton, Mesoplankton, Macroplankton, Particulate Organic Matter, Particulate Organic Matter river, Phytoplankton, Seagrass, Seagrass detritus, Sedimentary Organic Matter, Suspended Organic Matter, Suspended Organic Matter Sewage, Trapped Organic Matter).
**Sample**	***Phylum***	WoRMS standardised and up-to-date taxonomic classification at the Phylum level based on the taxonomic classification and nomenclature provided by the authors.
**Sample**	***Class***	WoRMS standardised and up-to-date taxonomic classification at the Class level based on the taxonomic classification and nomenclature provided by the authors.
**Sample**	***Order***	WoRMS standardised and up-to-date taxonomic classification at the Order level based on the taxonomic classification and nomenclature provided by the authors.
**Sample**	***Family***	WoRMS standardised and up-to-date taxonomic classification at the Family level based on the taxonomic classification and nomenclature provided by the authors.
**Sample**	***Genus***	WoRMS standardised and up-to-date taxonomic classification at the Genus level based on the taxonomic classification and nomenclature provided by the authors.
**Sample**	***Species***	WoRMS standardised and up-to-date taxonomic classification at the Species level based on the taxonomic classification and nomenclature provided by the authors.
**Sample**	***Sex***	Sex (i.e., M: male; F: female); for pooled samples, the information was reported when consistent within the pool.
**Sample**	***Maturity***	Functional indication of the life stage as provided by the authors (i.e.,18–24 months, Adult, Calf, Chick, Immature, Juvenile, Larvae, Larvae I, Larvae II, Larvae- Post flexion, Larvae- Pre flexion, Late-larva, Mature, Recruit, Sub-adult). For pooled samples, the information was reported when consistent within the pool.
**Sample**	***Size (cm)***	Length, mesh size (single value, mean value, range). When available, associated errors are provided in brackets.
**Sample**	***Size type***	Measured body part (i.e., Carapace length, Cephalothorax length, Curved carapace length, Fork length, Mantle length, Maximum shell length, Net mesh size - Pore/sieve size-serial filtration (derived-body size based on mesh/sieve size), Pre-anal length, Shell length, Standard Length, Straight fork length, Total length).
**Sample**	***Weight (g)***	Individual body wet weight (single value, mean value, range). When available, associated errors are provided in brackets.
**Sample**	***Tissue***	Type of analysed tissue (i.e., Blood, Body portion, DHGL - Digestive gland, Hepatopancreas, Liver), Eggs, Feather, Gills, Hair, Leaf, Muscle, Otolith, Rhizome, Whole organism).
**Sample**	***Pooled samples/replications***	N° of individuals or sampling replicates in aggregated samples. “na” was reported when this information was not reported.
**Sample**	***Sample delipidisation***	Sample pre-treatment leading to the removal of lipids (i.e., No lipids removal (N); Lipids removal (Y); C/N-based mathematically corrected when C/N < 3.5).
**Sample**	***Sample acidification***	Sample pre-treatment with acid to remove carbonates (i.e., No carbonate removal (N); Carbonate removal (Y).
**Sampling site**	***Latitude (decimal degrees)***	Sampling site’s latitude (exact or an approximation based on the geographical area description provided by the authors). When sampling points or areas were depicted in maps without a clear association with the specific samples, the geographical coordinates of a point selected within the spatial domain of the represented area were annotated. When samples were purchased from local markets and anglers, the coordinates of a randomly selected point in the fishing area were entered.
**Sampling site**	***Longitude (decimal degrees)***	Sampling site’s longitude (exact or an approximation based on the geographical area description provided by the authors). When sampling points or areas were depicted in maps without a clear reference to the specific samples, the geographical coordinates of a point selected within the spatial domain of the represented area were annotated. When samples were purchased from local markets and anglers, the coordinates of a randomly selected point in the fishing area were entered.
**Sampling site**	***IHO Sea Area***	IHO (International Hydrographic Organization) Sea Area of the sampling site (i.e., Adriatic Sea, Aegean Sea, Alboran Sea, Balearic (Iberian Sea), Ionian Sea, Ligurian Sea, Mediterranean Sea - Central Basin, Mediterranean Sea - Eastern Basin, Mediterranean Sea - Western Basin, Strait of Gibraltar, Tyrrhenian Sea).
**Sampling site**	***MSFD Subregion***	MSFD (Marine Strategy Framework Directive) Subregion of the sampling site (i.e., MAD: Adriatic Sea; MAL: Aegean-Levantine Sea; MIC: the Ionian Sea and Central Mediterranean Sea; MWE: Western Mediterranean Sea).
**Sampling site**	***FAO subarea***	FAO *(*Food and Agriculture Organization) subarea of the sampling site (i.e., Western Mediterranean, Central Mediterranean, Eastern Mediterranean).
**Sampling site**	***Sampling depth (m)***	Depending on the sampling methodology used, the depth at which samples were collected, the maximum depth at which the sample may have been collected, or the maximum depth at which vertical profile sampling was performed (i.e., single value, range, minimum depth, maximum depth, mean depth). Approximated values were reported for replicated samples; the sampling depth was reported as text when reported by the authors (e.g., surface).
**Sampling site**	***Anthropic pressure or natural conditions***	Type of anthropic or natural pressure present in the sampling area, as indicated by the authors (i.e., Desalination plants, Fish ponds in transitional waters, Harbour, Lagoon, Mariculture (fish cages), Military area, MPA, Mussel farm, Other, Protected area, Saltworks, Shellfish farm, Wild-river dilution zone). We enter “Natural conditions” when the source papers do not indicate the presence of specific environmental/anthropic pressures in the studied area; the class “other” includes samples taken from transplantation experiments and pollution gradients from point sources.
**Sampling time**	***Year***	The yearly period during which the sampling was conducted (single years, range of years).
**Sampling time**	***Season***	The seasonal time span in which the sampling was carried out (single quarters, whole year).
**C and N characterisation**	***δ***^***13***^***C (***‰ vs VPDB)	Carbon isotope ratio (single value, mean value, Bayesian posterior estimates): untreated samples, delipidized samples, lipid effect mathematically corrected.
**C and N characterisation**	***δ***^***13***^***C – error***	The error associated with the *δ*^13^C value.
**C and N characterisation**	***δ***^***13***^***C- error type***	Type of error associated with *δ*^13^C value (i.e., standard deviation, standard error, CV).
**C and N characterisation**	***δ***^***15***^***N (***‰ vs N_2_***)***	Nitrogen isotope ratio (single value, mean value, Bayesian posterior estimates).
**C and N characterisation**	***δ***^***15***^***N - error***	The error associated with the δ^15^N value.
**C and N characterisation**	***δ***^***15***^***N - error type***	Type of error associated with *δ*^15^N value (i.e., standard deviation, standard error, CV%).
**C and N characterisation**	***C/N***	The ratio of organic carbon to total nitrogen: untreated samples, delipidized samples, acidification, lipid effect mathematically corrected.
**C and N characterisation**	***C/N – error***	The error associated with the C/N value.
**C and N characterisation**	***C/N - error type***	Type of error associated with C/N value (i.e., standard deviation, standard error, CV%%).
**C and N characterisation**	**Notes**	Additional information and relevant details.
**Source**	***Bibliographic reference***	Reference details.

38 ISOMED parameters within the 6 data categories, i.e., DB compilation, sample, sampling site, sampling time, C and N characterisation, source: parameter short name and description. When no or ambiguous information was provided to fill the metadata fields, “Not applicable” (i.e., na) was entered in the cells.

## Metadata and Data Compilation

### Sample description

Each record entered into the database was associated with the original sample name used by the authors of the source paper (***Original sample name***) (e.g., Table 1). To ensure consistency in the descriptions of the samples, the nomenclature of both samples at the base of the trophic web (i.e., organic matter sources, primary producers, plankton, and meiofauna) and of higher-order consumers was standardized. For the former, each entry was assigned to a representative macrocategory (***Functional category***), intended to improve the practical use of the database. For the latter, based on the scientific name or taxonomic information provided by the source, an updated and standardised taxonomic classification was compiled by referring to the World Register of Marine Species (WoRMS; http://www.marinespecies.org/; ***Phylum, Class, Order, Family, Genus, Species***). The species were classified based on the most detailed taxonomic level mentioned in the respective manuscript.

When available, information about the variables ***Sex***, ***Maturity***, ***Size*** (in cm, as reported in the ***Size type***), and ***Weight*** (fresh wet weight, in g) was recorded for consumers. Finally, information was provided about sample preparation and pre-treatment prior to isotopic analysis. The last four columns provide insights into the analysed body part or tissue (***Tissue***), the representativeness of the samples (***Pooled samples/replications*)**, and any sample pre-treatments for removing lipids and carbonates (***Sample delipidisation, Sample acidification***). Lipids and carbonates can affect *δ*^13^C values^31,32^; therefore, some samples may have undergone delipidization or acid decarbonization before isotopic analysis. Alternatively, effect of lipids on *δ*^13^C values might have been corrected *a posteriori* through mathematical equations^31–35^. When authors reported both pre- and post-delipidisation *δ*^13^C values, we exclusively annotated the lipid-adjusted values.

### Sampling site and time description

The geographical coordinates of the sampling sites listed in the source papers for each data entry (converted to decimal degrees; ***Latitude***, ***Longitude***) were also reported. When the source papers report multipoint-sampling surveys within a limited area or broadly identify the study location (e.g., via toponymy), the midpoint coordinates of the indicated region were entered. Based on the geographical coordinates, the columns related to the ***IHO*** (***International Hydrographic Organization***) ***Sea Area***, **Marine Strategy Framework Directive** (**MSFD**) ***subregion***, and the ***FAO*** (**Food and Agriculture Organization**) ***subarea*** were compiled. Indication regarding the sampling depth was also annotated (***Sampling depth***).

We supplemented the sampling site description with information about the presence of anthropic pressures (e.g., the occurrence of fish farms, chemical pollution) or particular conditions (e.g., river dilution, protected areas) when considered relevant for interpreting the isotopic data (***Anthropic pressure or natural condition***). Source papers were designed for various distant research scopes and, therefore, they can intentionally focus on areas where specific sources and dynamics influence *δ*^13^C and *δ*^15^N values. This information was compiled based on the authors’ description of the sampling area.

Finally, records were complemented with the sampling time information when available (***Year, Season***). While the year was reported according to the format used in the source paper (i.e., single year, multiple years, ranges), the seasonal data were standardised into quarters.

### Isotopic characterisation

The *δ*^13^C and *δ*^15^N values were extracted from the papers’ main text, tables, graphs, or supplementary materials; the associated errors were entered into separate columns, with the indication of the error type (***δ***^**13**^**C,** ***δ***^***13***^***C*** – ***error***, ***δ***^**13**^**C** - ***error type***, ***δ***^**15**^**N,** ***δ***^**15**^**N** – ***error***, ***δ***^**15**^**N** - ***error type***). We also collected the organic carbon (after acidification, specified in the database) to total nitrogen ratio (C/N) when available (***C/N***, ***C/N*** – ***error***, ***C/N*** - ***error type***). Whenever relevant, additional details were provided in the last column ***Notes***.

### Spatial and taxonomic coverage

Once subjected to quality control procedures, the final dataset consisted of 4,959 georeferenced records of occurrence spread throughout the Mediterranean Sea, spanning a period of 35 years (1983–2018). It is freely accessible for download from SEANOE, a permanent repository hosting sea-related open data, and it adheres to the FAIR principles of Findability, Accessibility, Interoperability, and Reusability of data.

**Fig. 1:**
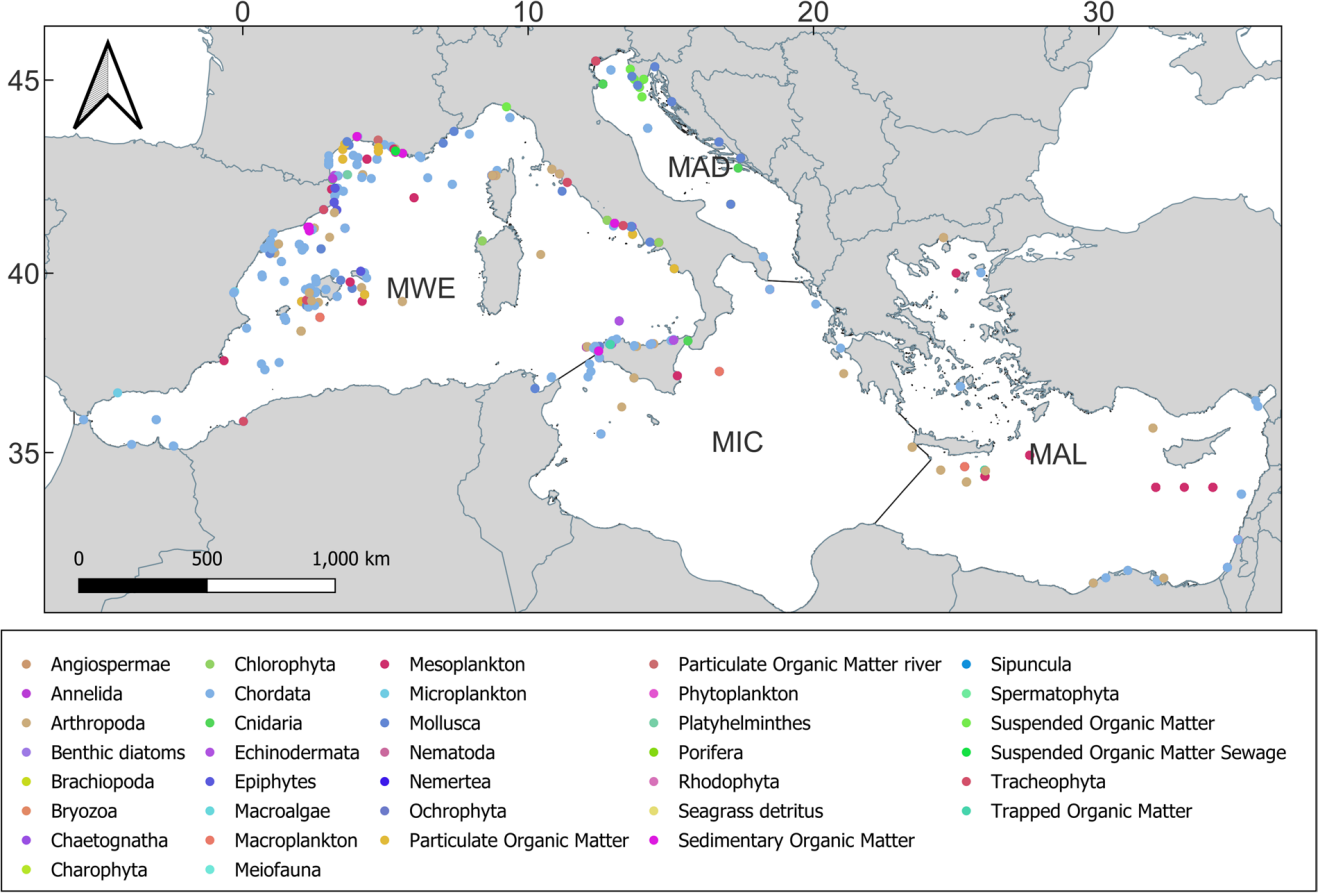
Maps of the georeferenced records of ISOMED for the 4 Mediterranean subregions according to MSFD division: (**a**) Western Mediterranean Sea; (**b**) Adriatic Sea, Ionian Sea and Central Mediterranean Sea (the two subregions are represented together); (**c**) Aegean and Levantine Sea.

Most records (n = 3,674) were referred to the Western Mediterranean subregion, followed by the Adriatic Sea (n = 619), the Aegean-Levantine Sea (n = 431), and the Ionian Sea and Central Mediterranean Sea (n = 235) (e.g., Fig. 1).

**Fig. 2:**
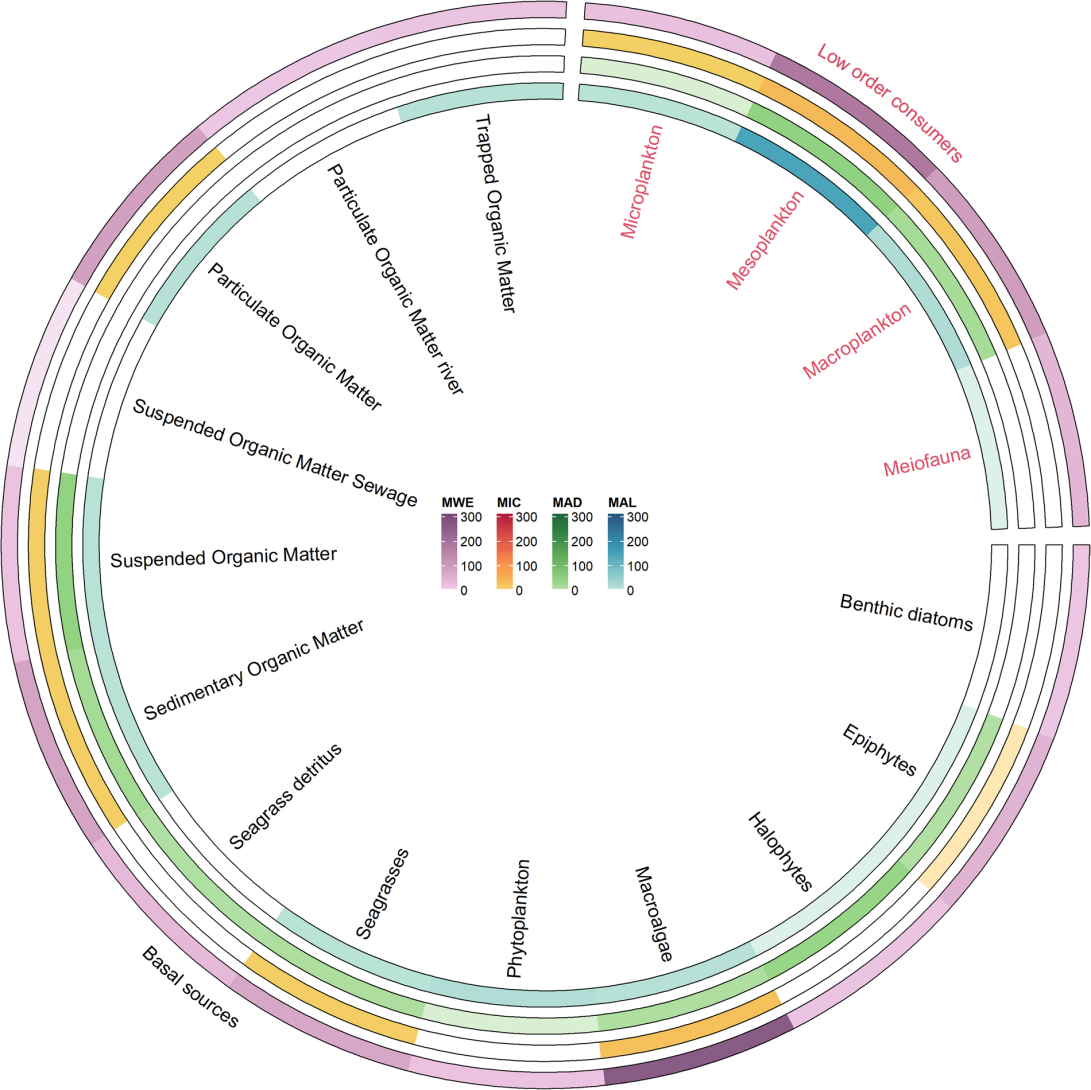
Circular heat map showing the frequency of isotopic data available for basal sources and low-order consumers organised into functional categories. Each ring corresponds to a different MSFD subregion (MWE: Western Mediterranean Sea subregion; MAD: Adriatic Sea; MAL: Aegean-Levantine Sea; MIC: Ionian Sea and Central Mediterranean Sea). The scale of colours indicates the number of data available.

**Fig. 3:**
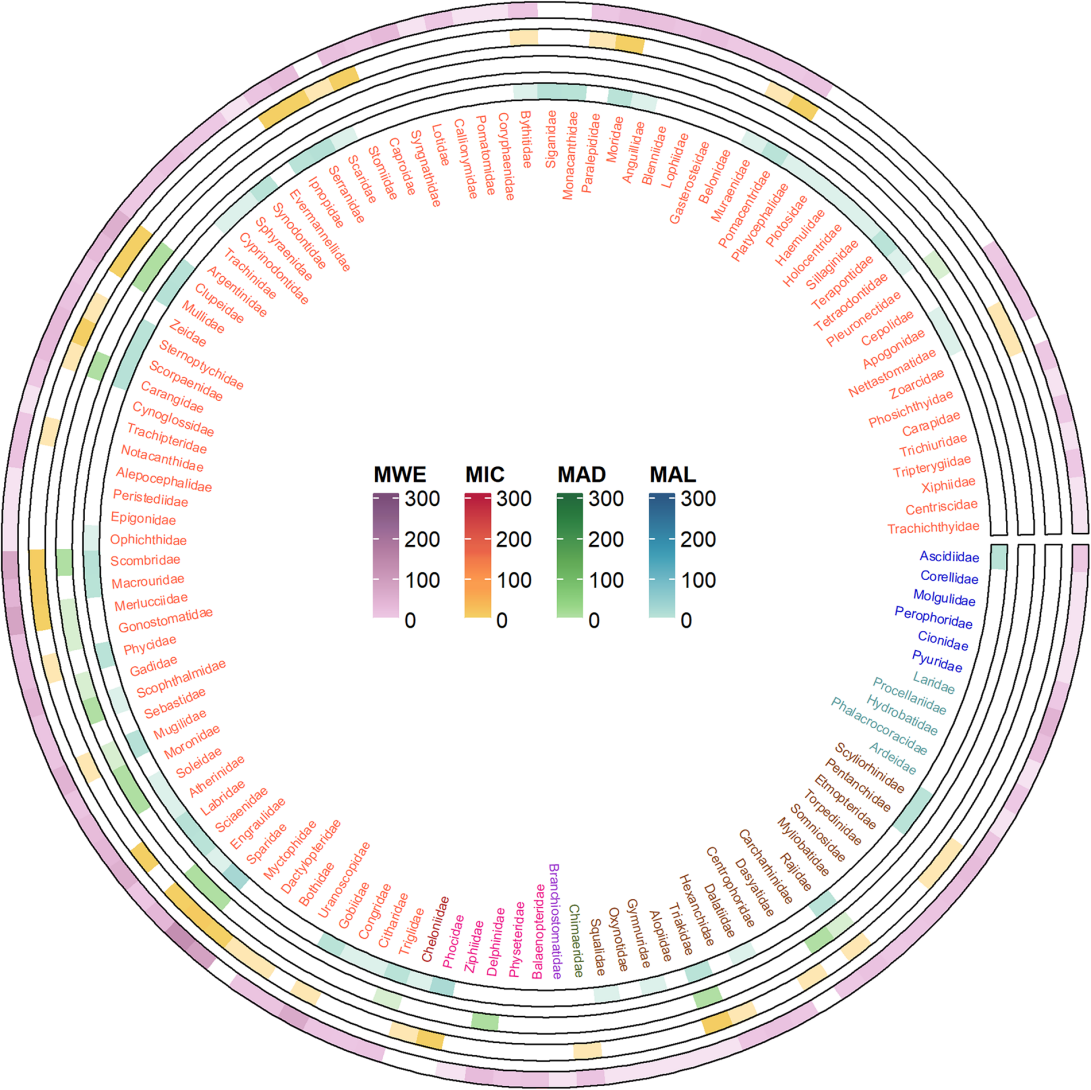
Circular heat map showing the frequency of isotopic data available for higher order consumers: Chordata. Colours refer to different classes. Each ring corresponds to a different MSFD subregion (MWE: Western Mediterranean Sea; MAD: Adriatic Sea; MAL: Aegean-Levantine Sea; MIC: the Ionian Sea and Central Mediterranean Sea; MWE: Western Mediterranean Sea). The scale of colours indicates the number of data available.

The dataset encompasses data covering the entire food web, ranging from basal sources (e.g., Suspended Organic Matter, Sedimentary Organic Matter, phytoplankton, or macrophytes, as shown in Fig. 1) to marine mammals (e.g., the monk seal, *Monachus monachus*) and birds (e.g., the Scopoli’s shearwater, *Calonectris diomedea*). Data concerning the basal source and lower-order consumers accounts for 29% of entries (1,429 records), while that referring to higher-order consumers makes up the remaining 71% (3,530 records). The distribution of records across key macrocategories and phyla within these two groups is illustrated in Figs. 2 and 3, respectively.

As presented in Fig. 2, ISOMED describes the base of the Mediterranean food web by incorporating 593 data points of various size-range planktonic organisms, along with 836 records referring to plant and algae species (both as alive organisms and detritus), as well as several types of organic matter samples (e.g., suspended, particulate organic matter, trapped organic matter). The majority of this data refers to the Western Mediterranean Sea subregion (MWE, 67%), with smaller proportions referring to the Aegean-Levantine Sea (MAL, 14%), the Adriatic Sea (MAD, 12%), and the Ionian Sea and Central Mediterranean Sea (MIC, 7%).

**Fig. 4:**
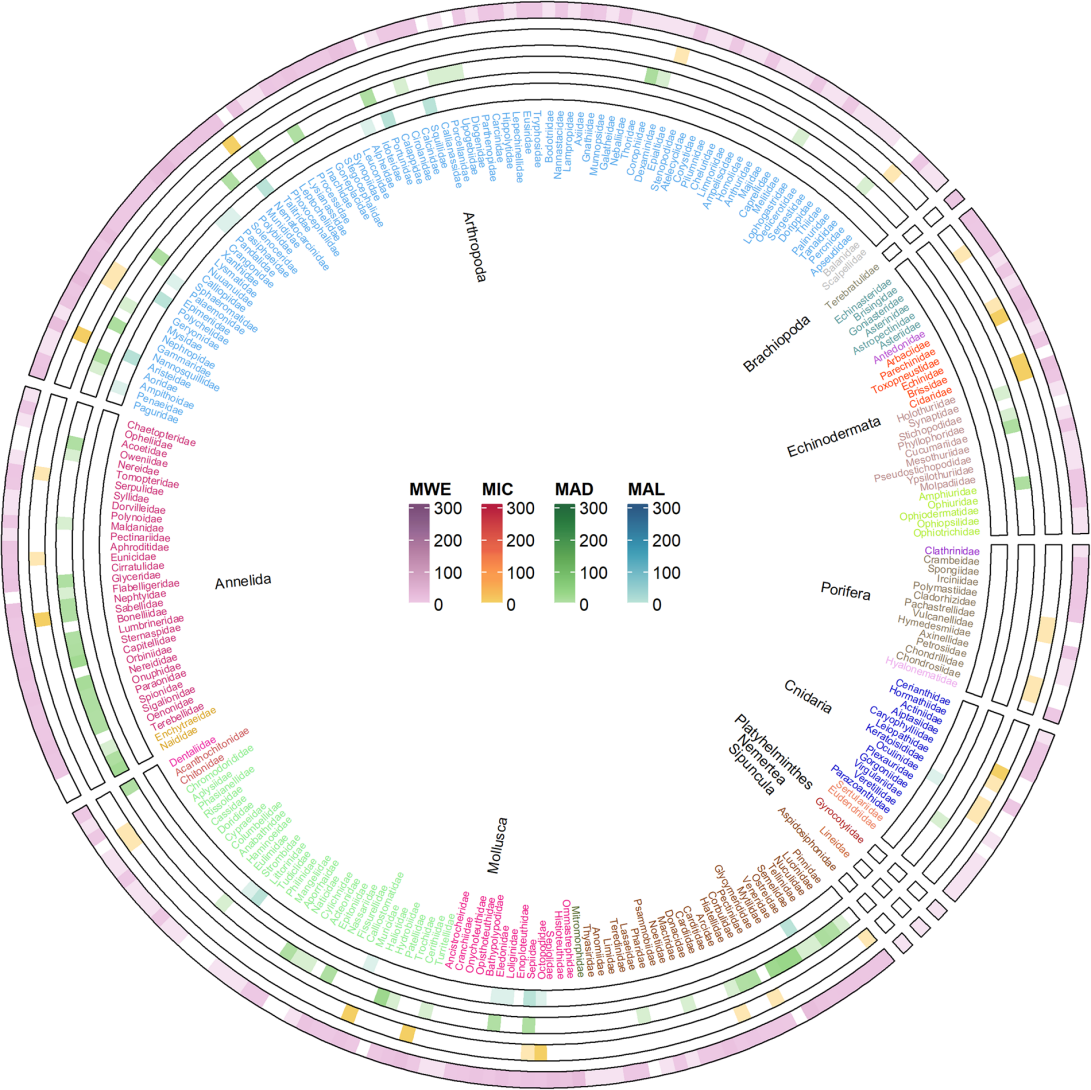
Circular heat map showing the frequency of isotopic data available for higher order consumers, organised into Phyla: invertebrates. Within each Phylum, colours refer to different classes. Each ring corresponds to a different MSFD subregion (MWE: Western Mediterranean Sea; MAD: Adriatic Sea; MAL: Aegean-Levantine Sea; MIC: the Ionian Sea and Central Mediterranean Sea; MWE: Western Mediterranean Sea). The scale of colours indicates the number of data available.

Concerning the higher order consumers’ data, a slight majority of data refer to Chordata (1854 records, 128 families; Fig. 3), whereas invertebrates accounted to 1657 records (Fig. 4), with records mostly belonging to Arthropoda (659 records, 88 families) and Mollusca (551 records, 75 families). The relative representation of these phyla is quite similar in the Western Mediterranean Sea (MWE) and in the Ionian Sea and Central Mediterranean Sea (MIC), with Chordata accounting for 57% and 62%, and Mollusca for 13% and 11%, respectively. Additionally, in the Adriatic Sea (MAD) and Aegean-Levantine Sea (MAL), the relative abundance of data for Chordata is 10% and 77%, 21% and 10% for Arthropoda, and 40% and 7% for Mollusca. Less than 1% of records are available for five phyla, cumulatively accounting for 1.4% of higher-order consumer records. These phyla are Sipuncula, Brachiopoda, Nemertea, Platyhelminthes, and Porifera.

## Data Records

The ISOMED database (version V1.0) is available for download from SEANOE as a single.xlsx file (Excel matrix) and can be accessed at: 10.17882/100661.

The file comprises i) a readme file, which provides a general explanation of the dataset, ii) a synthesis table (number of records by major taxa), iii) the ISOMED database and iv) a table with all the details of references to further facilitate their use and retrieval from original papers by researchers. Column headings refer to the 38 ISOMED parameters as described in Table 1.

## Technical Validation

Once compiled, the database was consolidated by performing basic quality controls to eliminate possible duplicate entries, spelling errors, typos, and inconsistencies.

*δ*^13^C and *δ*^15^N data were then visually inspected to identify possible values far outside their expected range. Clear outliers were verified by checking them in the source paper to avoid transcription errors. Uncertain records (e.g., evident typos in the original papers) were deleted entirely.

When no or ambiguous information was provided to fill the metadata fields, “Not applicable” (i.e., na) was entered in the cells.

Finally, all data records were verified by a peer-review process conducted by SEANOE.

## Usage Notes

The current version of the ISOMED database is v1.0. The database will be updated in the next few years with data from the literature, raw data, and analytical outputs emerging from different projects in which the authors are participating. Expected additions include expanding the taxonomic and geographic coverage of the database and increasing the sample size with data from recent years.

## Data Availability

The ISOMED database is available at 10.17882/100661^36^.

## Code availability

No custom code was used during the compilation of the dataset. Maps were created using QGIS 3.42. Figures were generated using the R packages circlize^37^ and ComplexHeatmap^38^.
